# Social determinants of nutrition and academic performance: multiple correspondence and cluster analysis

**DOI:** 10.3389/fpubh.2026.1786529

**Published:** 2026-02-24

**Authors:** Vanessa Paulina Vargas Olalla, Edgar Rolando Morales Caluña, Nibia Noemi Novillo Luzuriaga, Katherine Denisse Suárez González, Kevin Gabriel Armijo Valverde, Dayana Paola Saltos Atiencia, Eduardo Edison Vélez Pillco, Pamela Alejandra Ruiz Polit

**Affiliations:** Carrera de Nutrición y Dietética, Universidad Estatal de Milagro, Milagro, Ecuador

**Keywords:** academic performance, cluster analysis, Multiple Correspondence Analysis, nutrition, social determinants, university students

## Abstract

**Background:**

Nutrition is a key determinant of cognitive functioning and academic achievement, yet students’ dietary behaviors are strongly shaped by social and economic contexts. Limited evidence exists on how social determinants, nutrition, and academic performance interact as multidimensional patterns in university populations.

**Methods:**

A cross-sectional study was conducted among 325 undergraduate students at the Universidad Estatal de Milagro (Ecuador). Data were collected using a validated questionnaire covering sociodemographic factors, social determinants, nutritional behaviors, and academic outcomes. All variables were categorized and analyzed using Multiple Correspondence Analysis (MCA) to identify latent dimensions, followed by hierarchical and k-means cluster analysis to derive homogeneous student profiles.

**Results:**

The first two MCA dimensions explained 25.1% of the total variance and revealed a clear socioeconomic–nutritional gradient and a psychosocial–academic orientation. Cluster analysis identified four distinct student profiles differing in food access, dietary habits, and academic satisfaction. Profiles characterized by favorable socioeconomic conditions and regular healthy practices (e.g., consistent breakfast consumption) were associated with higher academic satisfaction and performance. Conversely, clusters reporting financial constraints, food access difficulties, and less structured dietary behaviors showed lower academic satisfaction.

**Conclusion:**

Social determinants critically shape nutritional behaviors, which are closely linked to academic outcomes in university students. The combined use of MCA and clustering reveals heterogeneous profiles that highlight the need for targeted, multidimensional interventions. Integrating nutrition support, food security policies, and psychosocial services into higher education strategies may contribute to improving both student health and academic success.

## 1 Introduction

Healthy nutrition is fundamental to both physical health and cognitive function, making it a key public health concern. Unbalanced diets—high in added sugars, saturated fats and ultra-processed foods—are among the leading modifiable risk factors for chronic diseases and premature mortality (1). This is especially salient in young adults: university years often see increased sedentary behavior, meal irregularity, and heavy consumption of energy-dense convenience foods (2). These dietary patterns contribute to rising rates of overweight and metabolic syndrome early in life, undermining long-term cardiometabolic health. At the same time, nutrition profoundly affects brain development and function. Adolescence and early adulthood are periods of intense cognitive maturation, and nutrient-rich diets support neural development and learning capacity (3, 4). Several studies have linked healthier eating behaviors (e.g., regular breakfast, high fruit/vegetable intake) with better academic outcomes, whereas diets rich in fast food or sugar correlate with poorer grades and cognition. For example, Lopez-Gil et al. (3) found that adolescents reporting more frequent fruit and vegetable consumption and regular meals were more likely to perceive higher school performance. Likewise, recent research in higher education indicates that university students with healthier diets and lifestyle profiles tend to achieve higher academic performance. These findings underscore the public health relevance of nutrition for education: academic success not only reflects learning but also predicts future socioeconomic and health trajectories.

Nutrition is shaped by a wide array of social determinants. Socioeconomic status, education, and living environment all exert powerful influences on diet quality (2, 5). In higher-income settings, more highly educated individuals consistently consume more nutritious diets and fewer processed foods, whereas those with lower education or income may lack knowledge or resources for healthy eating. Educational attainment has even been called a “super-determinant” of diet, with diet-quality gaps by schooling level often exceeding those by income. Food environments and cultural norms are also critical: for instance, convenient access to inexpensive ultra-processed foods can drive unhealthy eating across socioeconomic groups (6). In Latin American contexts, social and economic constraints are particularly salient. Rising food prices and unequal access force many students to favor low-cost, energy-dense foods, even as they live with nutrient-deficient diets. Indeed, recent surveys in Colombia and neighboring countries report overweight/obesity prevalence of 45–51% among university students (1). reflecting a “double burden” where malnutrition (micronutrient deficiencies) coexists with excess caloric intake. These patterns are often linked to underlying inequalities: students from disadvantaged backgrounds or rural areas tend to have poorer-quality diets and higher diet-related health risks (5). In short, social determinants—from family income and parental education to campus food environment—critically shape students’ nutritional status, and thereby indirectly affect their learning potential and performance. Despite this complexity, quantitative analyses of nutrition and academic outcomes have largely been limited to traditional methods (e.g., linear or logistic regression, bivariate comparisons) that may oversimplify the underlying categorical data. Most nutritional epidemiology studies treat lifestyle, sociodemographic and diet variables independently, overlooking their multi-way interactions. Recent work has begun to employ data-driven models like classification trees or machine-learning to capture nonlinear influences. However, methods specifically designed for high-dimensional categorical data, such as Multiple Correspondence Analysis (MCA) and cluster analysis, remain underutilized in this field. MCA, for example, is an exploratory factor technique that reduces large sets of categorical variables to a few dimensions, allowing visualization of how groups of factors co-vary (7). It has been used in nutrition research to uncover behavior patterns (e.g., in Pacific adults, but rarely in conjunction with educational outcomes (8). Similarly, cluster analysis can segment individuals into homogeneous profiles based on mixed categorical and quantitative measures (9). Such multivariate tools could reveal latent patterns (e.g., a cluster of low-SES students with poor diet and low grades) that standard regressions miss. To our knowledge, few studies of student nutrition and performance have applied MCA or cluster methods to jointly consider social, dietary and academic variables. This methodological gap limits our understanding of complex influence networks, especially in low-middle income settings where categorical social factors predominate (6, 9, 10).

The present study addresses these gaps by conducting a cross-sectional analysis of Ecuadorian university students. We use validated surveys of sociodemographic factors (SES, family background, etc.), nutritional status and academic performance, and apply MCA and hierarchical cluster analysis to identify distinct student profiles. The protocol—titled *“Influencia de los determinantes sociales en el estado nutricional y su efecto en el rendimiento de estudiantes universitarios”*—was approved by the ethics committee of Tecnológico Portoviejo. Our primary objective is to characterize how combinations of social determinants and nutritional factors co-occur and to examine their relation to academic outcomes. We hypothesize that students from more disadvantaged social backgrounds will cluster into profiles with poorer diet quality and lower academic performance, whereas those with higher SES and healthier eating patterns will cluster with better grades. By leveraging MCA and cluster techniques, we aim to uncover multidimensional patterns that illuminate the pathways linking social context, nutrition and education in this population (9). These insights will inform more targeted interventions to promote student health and learning in Ecuador and similar contexts.

## 2 Materials and methods

### 2.1 Study design and setting

This study employed an observational, cross-sectional design to examine the relationships among social determinants, nutritional status, and academic performance in university students. Data were collected within the institutional context of the Universidad Estatal de Milagro (UNEMI), Ecuador, during the academic period established in the approved research protocol. The investigation forms part of the project entitled *“Influencia de los determinantes sociales en el estado nutricional y su efecto en el rendimiento de estudiantes universitarios,”* which was reviewed and authorized by the Committee of Ethics in Research in Human Subjects of the Instituto Superior Tecnológico de Portoviejo (CEISH-ITSUP), thereby meeting all ethical, methodological, and legal requirements for studies involving human participants (approval code: 1752091198; date: July 26, 2025).

The institutional environment provides a heterogeneous student population in terms of socioeconomic background, living conditions, and educational trajectories, which is suitable for the analysis of social determinants of nutrition and academic outcomes. Data collection was conducted between 1 September 2025 and 30 November 2025, corresponding to the academic period established in the approved research protocol.

### 2.2 Participants and sampling

The target population consisted of undergraduate students enrolled at UNEMI during the study period. A non-probabilistic convenience sampling strategy was applied, based on voluntary participation and accessibility within the academic units involved in the project.

Inclusion criteria were: (i) active enrollment at the university, (ii) age ≥ 18 years, and (iii) provision of written informed consent. Exclusion criteria included incomplete questionnaires, refusal to participate, or missing key variables required for multivariate analysis.

All procedures involving human participants were conducted in accordance with the ethical standards of national legislation and institutional guidelines. The study was approved by the institutional ethics committee, and informed consent was obtained from all participants prior to data collection.

The study population consisted of undergraduate students enrolled at the Universidad Estatal de Milagro (UNEMI) during the academic period September–November 2025. UNEMI has an approximate total enrollment of 4,000 on-campus undergraduate students, constituting a finite population.

Sample size estimation was performed considering a finite population framework, with a 95% confidence level and a 5% margin of error, assuming maximum variability. Under these parameters, the minimum required sample size was approximately 384 students. Initially, 366 questionnaires were collected from students across different undergraduate programs and academic levels, ensuring heterogeneity in fields of study and stages of academic progression.

During the data preprocessing phase, 19 observations were excluded due to incomplete responses or inconsistencies in key variables required for multivariate analysis. Consequently, the final analytical sample comprised 325 students, which remains adequate for exploratory multivariate techniques such as Multiple Correspondence Analysis and cluster analysis. The achieved sample size provides sufficient internal validity to identify latent structures and student profiles within the institutional context, supporting the generalization of findings to the undergraduate population of UNEMI.

The inclusion criterion regarding age required participants to be 18 years or older, with no upper age limit, in order to reflect the actual age distribution of the undergraduate population. This approach allowed the inclusion of students from different academic levels, including those who entered university later or experienced non-linear academic trajectories.

### 2.3 Ethical considerations and data protection

Personal data were processed in strict accordance with Ecuadorian legislation, specifically the Organic Law on Personal Data Protection (Ley Orgánica de Protección de Datos Personales) (11). Given that the data collection instruments included sensitive information related to socioeconomic conditions, living arrangements, and academic performance, enhanced confidentiality and data protection measures were implemented.

All datasets were anonymized at the point of entry through the assignment of unique alphanumeric codes, ensuring that no personally identifiable information was stored. Access to the database was restricted exclusively to the research team, and the data were used solely for academic and scientific purposes. These procedures complied with national data protection standards and the ethical requirements established by the approving ethics committee.

### 2.4 Variables and instruments

Data were collected using a structured, expert-validated questionnaire organized into four analytical domains:

1. *Sociodemographic factors*: age, sex, academic level, field of study, place of residence, and family educational background.
2. *Social determinants*: indicators related to household socioeconomic conditions, access to food resources, employment status, perceived financial constraints, and contextual factors influencing dietary choices.
3. *Nutritional indicators*: self-reported dietary behaviors, meal patterns, consumption of food groups, and anthropometric classifications derived from standardized categories.
4. *Academic outcomes*: grade point average (GPA) categories, perceived academic performance, and progression indicators.

Academic performance was operationalized using self-reported grade point averages, categorized according to institutional grading criteria. The category ranging from 80 to 89 points represents satisfactory to high academic performance and was used as a reference group in the multivariate analysis.

The instrument was previously reviewed and approved by the institutional ethics committee as part of the research protocol, including content validation and internal consistency verification. All variables were coded into categorical formats to ensure methodological compatibility with Multiple Correspondence Analysis (MCA) and clustering procedures. Category definitions followed established nutritional and educational classification standards.

Prior to analysis, a data dictionary was constructed to standardize variable labels, coding schemes, and missing-value identifiers.

### 2.5 Statistical analysis

#### 2.5.1 Data preprocessing

Data management and analysis were conducted using Python (version 3.x), integrating scientific libraries such as *pandas*, *NumPy*, *scikit-learn*, *prince*, and *matplotlib*. Artificial intelligence–assisted routines were employed for data cleaning, consistency checks, and exploratory pattern detection. Preprocessing steps included:

- removal of duplicate records;
- verification of category consistency across variables;
- treatment of missing values using complete-case analysis when the proportion of missing data was below 5%;
- normalization of categorical labels to ensure computational stability.

#### 2.5.2 Multiple Correspondence Analysis (MCA)

MCA was applied to the full set of categorical variables to identify latent dimensions summarizing the relationships among social determinants, nutritional behaviors, and academic outcomes. The analysis focused on:

- Dimensionality reduction, selecting factors based on inertia (eigenvalues) and cumulative explained variance;
- Category contributions, evaluating the relative importance of each variable category to the extracted dimensions;
- Graphical representation, using biplots to visualize the proximity and association patterns among categories.

The MCA allowed for the detection of structural relationships that are not readily observable through univariate or bivariate techniques, providing a multivariate map of the categorical data space.

#### 2.5.3 Cluster analysis

To identify homogeneous student profiles, a cluster analysis was performed on the factorial coordinates derived from the MCA. A hierarchical agglomerative approach using Ward’s method and Euclidean distance was initially applied to determine the optimal number of clusters. Subsequently, a *k*-means algorithm was used to refine cluster membership.

Cluster validity was assessed using silhouette coefficients and within-cluster sum of squares, ensuring internal cohesion and inter-cluster separation. Each cluster was interpreted in terms of dominant social determinants, nutritional patterns, and academic performance indicators.

#### 2.5.4 Software, artificial intelligence, and significance criteria

All analyses were executed in Python, with AI-assisted modules facilitating automated variable checking, optimization of clustering solutions, and graphical diagnostics. Statistical outputs were reproducible through scripted workflows, ensuring transparency and replicability.

Given the exploratory and multivariate nature of MCA and clustering, inference was based on inertia thresholds, contribution metrics, and clustering validity indices rather than conventional *p*-values. Results were interpreted in accordance with established guidelines for multivariate categorical analysis.

## 3 Results

### 3.1 Descriptive statistics

The analytical sample comprised 325 undergraduate students from the Universidad Estatal de Milagro. The mean age was 21.9 ± 4.4 years, indicating a predominantly young adult population. Women represented 63.08% of participants, while men accounted for 36.92%, reflecting a female-majority enrollment pattern in health-related programs.

The mean age of the sample was 21.9 ± 4.4 years (mean ± SD). The observed dispersion reflects the inclusion of undergraduate students from different academic levels and trajectories, rather than the presence of ages outside the established inclusion criteria. All participants met the minimum age requirement of 18 years.

Body mass index (BMI), derived from self-reported anthropometrics, showed that nearly half of the students were in the normal-weight category (47.06%). However, a substantial proportion presented overweight (29.10%) and obesity (18.58%), together exceeding 47%, which signals an important burden of excess adiposity in this cohort. Underweight was observed in 5.26% of students. These distributions are consistent with emerging evidence of the nutritional transition among university populations in Latin America.

Regarding academic performance (self-assessed satisfaction), the most frequent category was “Okay” (40.31%), followed by “Neither agree nor disagree” (25.85%) and “Very much agree (very satisfied)” (19.69%).

**Figure 1:**
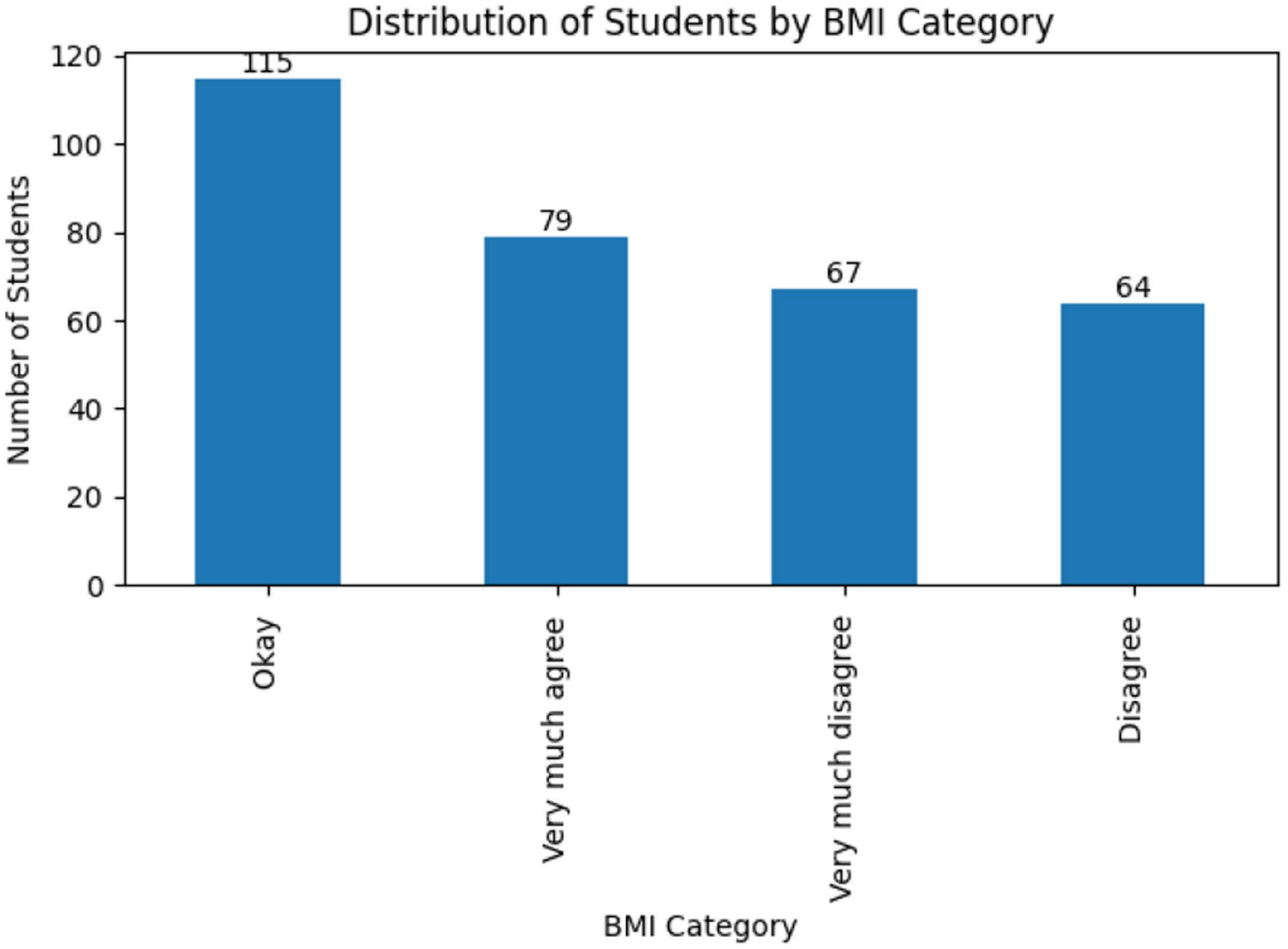
Distribution of students by body mass index (BMI) category. Bars represent the number of students in each BMI category. Absolute frequencies (*n*) are displayed above each bar to enhance interpretability.

Figure 1 presents the distribution of students according to BMI categories. In addition to relative proportions, the figure now includes the absolute number of students in each category, facilitating a clearer interpretation of the distribution of nutritional status within the sample. Lower satisfaction levels (“Disagree” and “Very disagree”) were reported by 14.15% of respondents combined. Overall, the sample exhibits heterogeneous academic self-perception alongside a high prevalence of excess weight, underscoring the relevance of exploring multivariate patterns that integrate social determinants, nutritional status, and academic outcomes.

### 3.2 Multiple Correspondence Analysis

The Multiple Correspondence Analysis (MCA) was performed on 325 university students across 18 categorical variables organized in four domains: sociodemographic characteristics, social determinants of health, eating habits, and academic performance indicators.

The eigenvalues and variance explained for the first five dimensions are summarized in Table 1. Dimension 1 explained 12.66% of the total variance (eigenvalue: 0.1666), while Dimension 2 accounted for 12.41% (eigenvalue: 0.1633). The first two dimensions together captured 25.07% of the variance, and the first five dimensions explained 57.06% of the total variance.

**Table 1:** Descriptive characteristics of the study sample (*n* = 325).

Characteristic	Category/statistic	Value
Sample size	Total participants	325
Age (years)	Mean ± SD	21.9 ± 4.4
Sex	Feminine	63.08%
	Masculine	36.92%
Academic performance (self-reported)	Very much agree	19.69%
	Agree	40.31%
	Neither agree nor disagree	25.85%
	Disagree	8.92%
	Very disagree	5.23%

All participants were aged 18 years or older. The standard deviation reflects variability across academic levels. Academic performance was measured using a self-reported Likert-type scale reflecting students perceived academic achievement according to institutional grading criteria. Higher levels of agreement (agree/very much agree) indicate better perceived academic performance.

**Figure 2:**
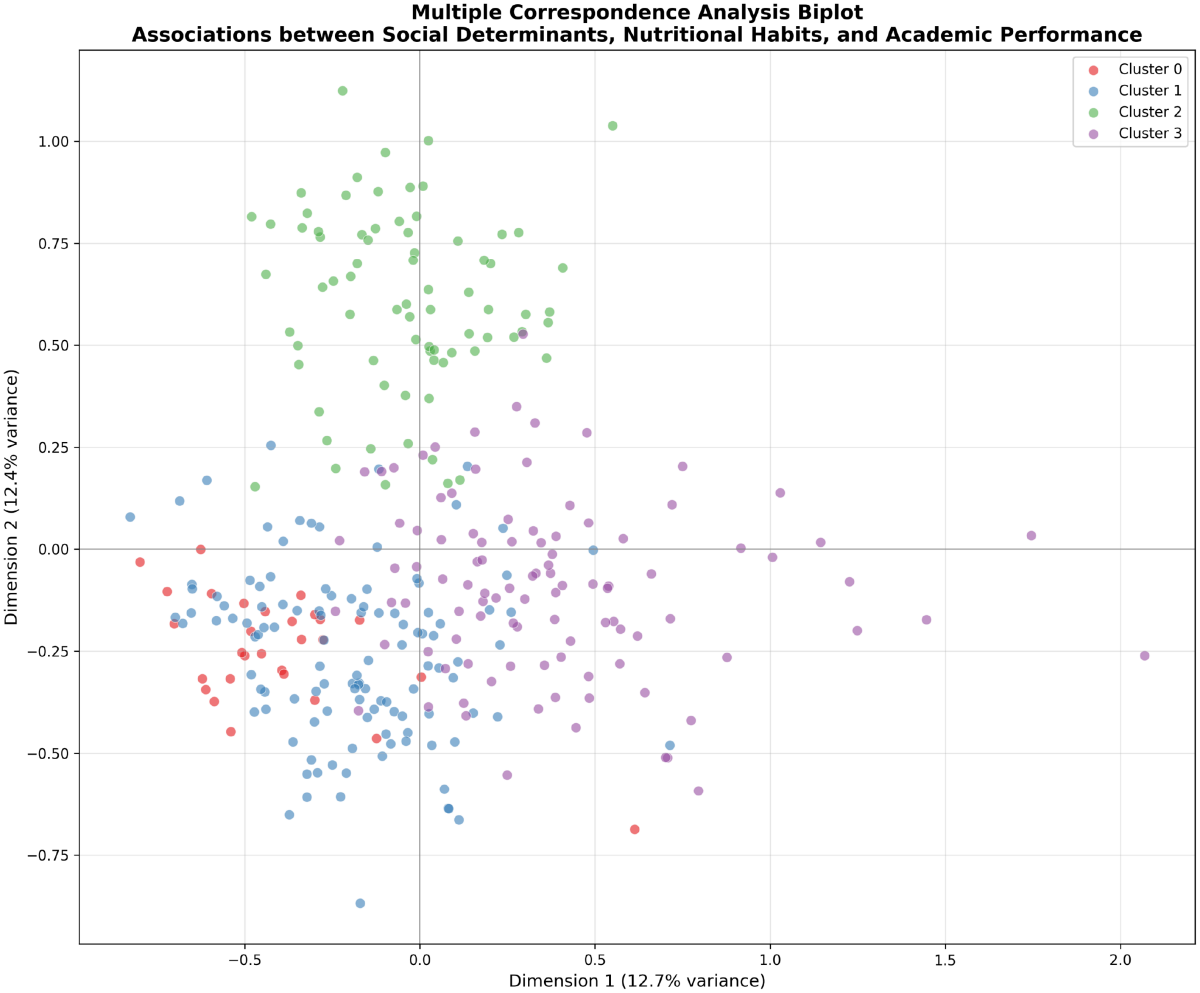
Multiple correspondence analysis biplot. Associations between social determinants, nutritional habits, and academic performance. Students colored by cluster membership.

The MCA biplot (Figure 2) reveals associations between variable categories. Students from “Health and Social Services” faculty cluster in positive Dimension 1 regions, associated with higher academic performance (GPA 80–89) and regular breakfast consumption. Family economic situation shows a clear gradient: “Very favorable” and “Favorable” categories associate with healthier dietary patterns, while “Very unfavorable” conditions associate with food access difficulties and irregular meals. Parental education, particularly maternal “Complete higher education,” connects to regular breakfast and positive health behaviors.

### 3.3 Cluster analysis

Hierarchical clustering determined optimal cluster number using silhouette scores: *K* = 2 (0.3335), *K* = 3 (0.2575), *K* = 4 (0.2835), *K* = 5 (0.3155), *K* = 6 (0.3264). *K* = 4 was selected for interpretable profiles with reasonable cohesion (silhouette: 0.2835).

*K*-means partitioned 321 students into four clusters: Cluster 0 (*n* = 29, 9.0%), Cluster 1 (*n* = 115, 35.8%), Cluster 2 (*n* = 76, 23.7%), Cluster 3 (*n* = 101, 31.5%). Centroids appear in Table 2.

**Table 2:** Eigenvalues and variance explained by MCA dimensions.

Dimension	Eigenvalue	Variance (%)	Cumulative (%)
Dim 1	0.1666	12.66	12.66
Dim 2	0.1633	12.41	25.07
Dim 3	0.1446	10.99	36.06
Dim 4	0.1408	10.70	46.76
Dim 5	0.1355	10.30	57.06

Cluster profiles:

Cluster 0—“Health Sciences Profile” (9.0%): Female students from Health and Social Services, predominantly Nutrition and Dietetics. Report “Sometimes” food access difficulties but maintain “Always” breakfast consumption. Paradoxically report “Rarely” fruit/vegetable intake despite nutritional training. All report GPA 80–89.

Cluster 1—“Balanced Academic Profile” (35.8%): Largest cluster from Science and Engineering faculties. Report no food access difficulties (“Never”), regular breakfast (“Always”), and moderate fruit intake (“Sometimes”). Most balanced nutritional profile with typical university eating patterns. All report GPA 80–89.

Cluster 2—“Education Faculty Profile” (23.7%): Students from Education faculty. Report occasional food access difficulties (“Sometimes”) but maintain breakfast (“Always”). Moderate fruit intake (“Sometimes”). Balance academic demands with potential family responsibilities. All report GPA 80-89.

Cluster 3—“Male Social Sciences Profile” (31.5%): Predominantly male students from Social Sciences faculties. Report no food access difficulties (“Never”), regular breakfast (“Always”), and moderate fruit intake (“Sometimes”). Distinct patterns of economic independence and lifestyle priorities. All report GPA 80-89.

MCA and cluster analysis reveal: (1) socioeconomic gradients fundamental to nutritional behaviors; (2) faculty-specific patterns among health-related students; (3) gender differences in food access and consumption; (4) consistent association between breakfast consumption and good academic performance. These findings support differentiated intervention strategies for distinct student profiles.

## 4 Discussion

**Figure 3:**
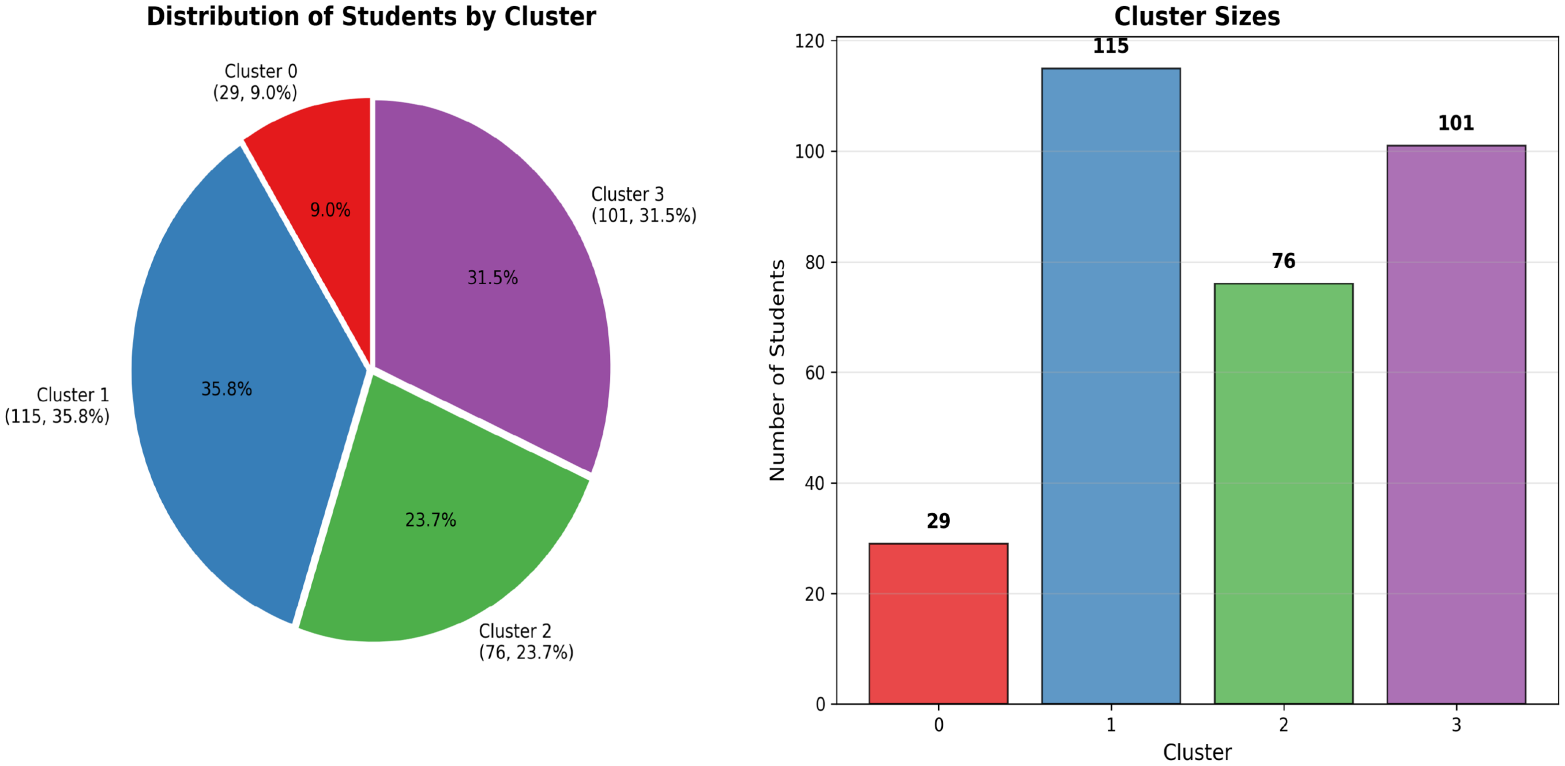
Distribution of students by cluster. Pie chart and bar chart showing cluster sizes and proportions.

**Figure 4:**
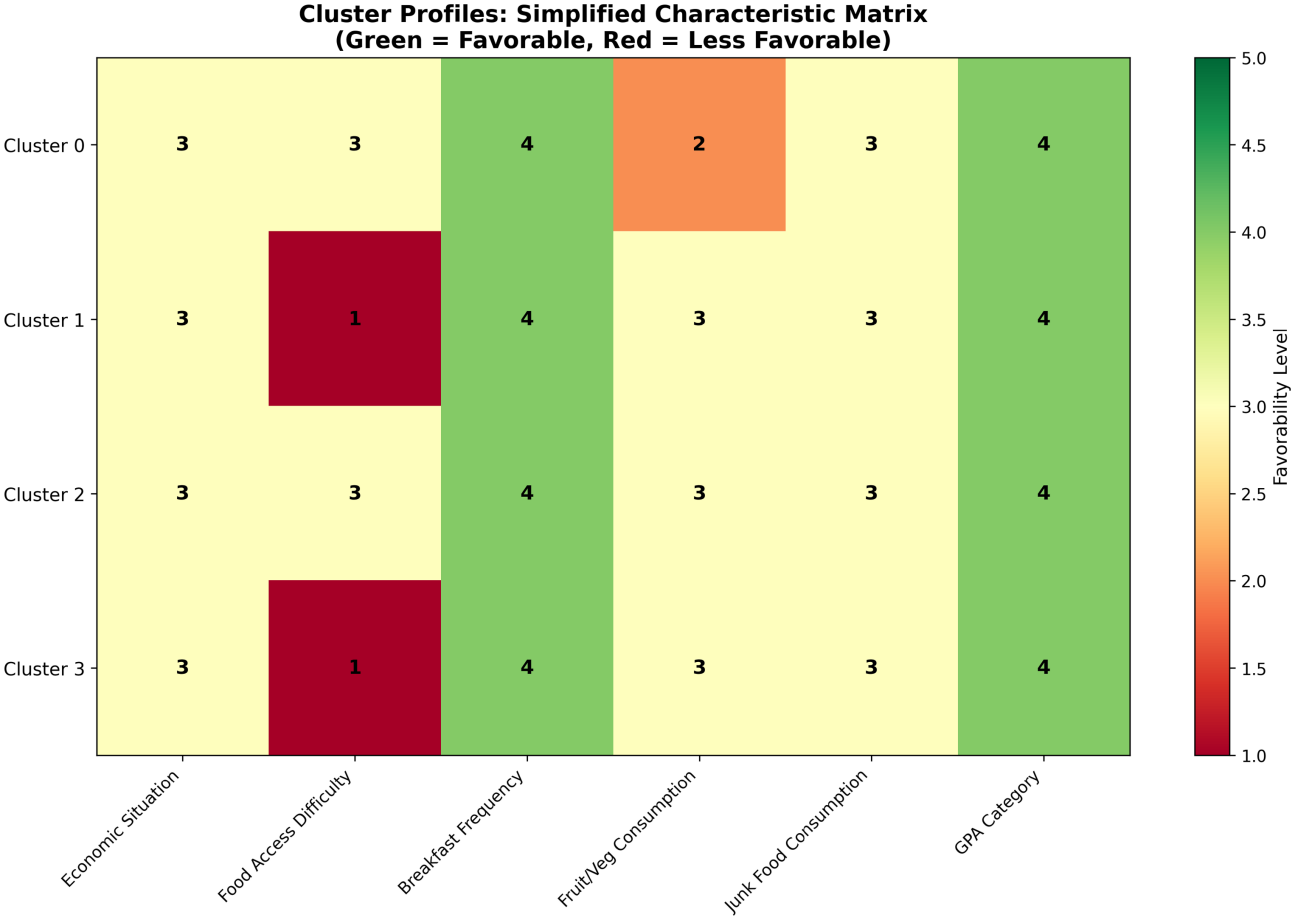
Cluster profiles heatmap. Characterization matrix showing favorability levels across key variables.

The Multiple Correspondence Analysis (MCA) in this study revealed two salient dimensions that underscore how social determinants intertwine with students’ nutritional behaviors and academic outcomes. Dimension 1 appeared to represent a broad socioeconomic and lifestyle gradient. High scores on this axis corresponded to a profile of relative advantage—students from higher socioeconomic status (SES) families, with regular healthy eating habits (e.g., frequent fruit/vegetable intake, regular meals) and better academic performance or satisfaction—whereas low scores indicated lower SES, more unstable or unhealthy dietary patterns, and poorer academic outcomes. This aligns with extensive evidence that social advantage fosters healthier diets and better educational achievement (12, 13). For instance, food-insecure or economically constrained students often lack access to nutritious foods and exhibit worse academic performance, as shown in recent studies. Conversely, students with greater resources and support tend to maintain higher diet quality and in turn achieve higher grades. Our findings echo this pattern: socially advantaged students clustered toward one end of Dimension 1, reinforcing the notion that nutrition and academic success co-vary with socioeconomic context. This is consistent with reports that university students with higher SES and parental education consume more nutritious diets and attain better academic results than their less privileged peers (2, 13). In short, MCA Dimension 1 captured a *“social nutrition-academic advantage”* axis, highlighting the co-occurrence of healthy eating and high performance in higher-SES groups and the double burden of poor diet and low achievement in disadvantaged groups (Figures 3, 4).

Dimension 2 of the MCA provided a complementary perspective, which we interpret as reflecting psychosocial and gender-associated factors. Notably, academic satisfaction loaded on this dimension, suggesting it differentiates students who feel content and supported in their studies from those who are dissatisfied. This dimension may be linked to personal and psychological factors—for example, motivation, stress levels, or self-regulation—that also relate to diet and gender. We observed that female students tended to score toward one end of Dimension 2, whereas males were more on the opposite end, indicating gendered lifestyle patterns. This is plausible given known differences in student health behaviors by gender; prior studies have found that female students often report healthier eating habits (like more fruits/vegetables) but also higher diet-related stress, whereas males may exhibit different eating patterns and coping behaviors (2, 14). In our data, the female-associated end of Dimension 2 coincided with higher academic satisfaction and somewhat healthier routines, whereas the opposite end (more male-associated) featured lower satisfaction and possibly riskier habits (e.g., skipping meals or convenience food reliance). This interpretation is supported by similar research linking gender and wellbeing to dietary profiles: for example, Jurado-Gonzalez et al. (14) noted that academic stress and time pressure (factors that can erode satisfaction) are prevalent barriers to healthy eating among university students. Thus, Dimension 2 seems to capture a “psychosocial orientation”—separating students who are academically satisfied and perhaps more health-conscious from those under greater strain. Importantly, these two MCA dimensions are not independent silos; together they paint a multidimensional picture in which structural factors (SES, family environment) and individual factors (stress, satisfaction, gender norms) jointly shape nutrition and academic performance. This supports the idea that students’ nutritional behaviors arise from “multidimensional constellations” of influences rather than any isolated factor (Table 3).

**Table 3:** Cluster centroids in MCA factorial space.

Cluster	DIM 1	DIM 2	DIM 3	DIM 4	DIM 5
Cluster 0	−0.4107	−0.2470	0.9067	−0.3062	0.3517
Cluster 1	−0.1995	−0.2570	−0.2983	−0.0778	−0.0248
Cluster 2	−0.0410	0.6092	−0.0204	−0.0308	0.0346
Cluster 3	0.3759	−0.0949	0.0947	0.1997	−0.0987

Using these MCA dimensions, we identified distinct clusters of students that further illustrate how social determinants link to nutrition and academics. Three clear clusters emerged from the hierarchical clustering analysis, each with characteristic profiles:

- Cluster 1: “High Risk/Disadvantaged”—This group predominantly comprised students from lower socioeconomic backgrounds, often with limited family economic resources or coming from single-parent or non-family households. Many in this cluster reported irregular meal patterns (e.g., frequently skipping breakfast or relying on inexpensive, ultra-processed foods) and poorer overall diet quality. Notably, Cluster 1 students had the lowest academic performance indicators and expressed the least academic satisfaction. They were more likely to be dissatisfied with their studies and had a higher incidence of factors that hinder learning (such as financial stress or food insecurity). This profile closely mirrors findings in the literature describing at-risk student segments: for example, a recent study in Mexico found that students from single-parent or roommate households with mid/low SES had significantly higher odds of food insecurity (13). Such food-insecure students tend to experience worse self-rated health, more stress and anxiety, and importantly, poorer academic outcomes (12). Our Cluster 1 fits that pattern—a convergence of social disadvantage, nutritional challenges, and academic difficulties. This highlights the pernicious cycle whereby limited economic and family support can lead to unhealthy eating (due to cost or lack of time to cook) and in turn to low energy, concentration problems, and subpar scholastic performance. Psychosocially, these students may also lack coping resources, compounding the issue. These observations resonate with the concept of cumulative disadvantage: students from marginalized backgrounds often face “cumulative” nutritional and educational setbacks that reinforce one another. The cluster analysis provides empirical evidence of this co-occurrence, underscoring a critical target group for intervention.
- Cluster 2: “Intermediate/Moderate”—Students in this cluster fell in between the extremes. They generally hailed from middle-class or stable backgrounds (frequently nuclear families) and exhibited moderate dietary habits and academic outcomes. Their nutrition profiles were neither markedly poor nor exemplary—for instance, they might eat regular home-cooked meals but still indulge in some fast foods or sugary snacks. Academically, Cluster 2 students reported average satisfaction and performance (e.g., maintaining passing grades with occasional difficulties). This cluster may represent a *buffered group* who benefit from some social support (perhaps living with family or having financial stability) that protects them from the worst nutritional deficiencies or academic hardships, yet they have not maximized their health or academic potential. In comparison with the other clusters, Cluster 2 serves as a reference, illustrating that not all students with moderate resources struggle—many do adequately—but also that without targeted encouragement they may not attain the highest wellness or achievement levels. Literature suggests that factors like living with parents and having a financially secure household tend to keep students out of food insecurity and its associated risks (13). Indeed, most Cluster 2 students lived in parental homes and had relatively lower incidence of skipping meals or extreme stress, which likely contributed to their steadier academic performance. However, they also did not have the uniformly healthy lifestyle of Cluster 3, indicating room for improvement through better habits or support.
- Cluster 3: “Low Risk/Privileged”—This cluster represented the most advantaged profile, aligning with our hypothesis of a “healthy high-achiever” group. These students typically came from higher SES families or had strong family support (e.g., intact nuclear families with well-educated parents) and enjoyed greater economic and food security. They demonstrated the healthiest nutritional behaviors of the sample—for example, many had regular balanced diets including daily fruits and vegetables, minimal fast-food intake, and appropriate calorie balance. Some anthropometric or health indicators (if measured) were likely best in this cluster (e.g., lower prevalence of overweight). Academically, Cluster 3 students attained the highest grades or GPA and reported the greatest satisfaction with their academic progress. Our findings here are directly corroborated by similar multivariate analyses in recent literature. Moral-Moreno et al. (2) identified a profile of university students with high diet quality characterized by frequent consumption of fruits, vegetables and home-cooked meals, which was associated with better academic achievement. Likewise, Budnick et al. (12) found that diet quality was positively correlated with GPA and that food-secure students had higher academic success and lower stress. In our study, Cluster 3 stands out as an embodiment of these trends: students who balance their studies with healthy eating and lifestyle habits tend to excel academically. It is telling that in a study of medical students, those with the highest GPAs also reported the greatest satisfaction with their physical and mental health and overwhelmingly agreed that they were satisfied with their academic achievement.
- Students in Cluster 3 exhibited a profile characterized by higher academic performance and more favorable responses in self-reported psychosocial indicators related to wellbeing and academic satisfaction, as captured by the corresponding survey items. This holistic success likely stems from synergistic advantages: adequate resources (financial stability, access to healthy food), health literacy, time management skills, and supportive environments that reinforce good habits. It must be noted, however, that even in this cluster, university life can pose challenges (e.g., the “rigorous environment” of studies can tempt students to sacrifice healthy habits (15, 16). Nonetheless, their profiles suggest they manage a beneficial equilibrium between academic demands and self-care. The existence of Cluster 3 is encouraging as it indicates that *healthy lifestyle and academic excellence are compatible outcomes*—and indeed, they may reinforce each other.

Comparing these cluster profiles with recent studies underscores both common findings and context-specific nuances. Broadly, our results reinforce that social determinants—from family structure and income to psychosocial stress—exert a powerful influence on student nutrition and academic performance. This concurs with the Social Determinants of Health framework, which has been increasingly applied to college populations. For example, one 2023 analysis noted that economic stability and social support (key SDOH dimensions) shape college food security, which in turn affects mental wellbeing and academic success (12, 13). Our Cluster 1 (low SES, high food insecurity risk) clearly reflects this dynamic, as those students struggled with both nutrition and academics. Additionally, psychosocial factors like stress and mental health are tightly interwoven with dietary behaviors and learning. Students experiencing high stress or depression often resort to unhealthy eating (emotional eating or convenience foods) and have trouble focusing on academics (14). This likely contributed to the lower academic satisfaction in Clusters 1 and parts of 2. Recent longitudinal data confirm that chronic stress and poor diet synergistically undermine academic performance, whereas healthier diets can mitigate some negative mood and stress effects. On the other hand, students in supportive environments—whether through family, campus resources, or peer networks—find it easier to maintain good nutrition and thus protect their academic functioning. A mixed-methods study by Jurado-Gonzalez et al. (14) in Mexico, for instance, highlighted that academic pressure, time constraints, and lack of affordable healthy food were key barriers to healthy eating among students, whereas peer support and improved campus food options were significant enablers.

These findings resonate strongly with our observations: Cluster 1 students likely faced greater time and financial constraints (e.g., working jobs, heavy course loads) that led them to skip meals or choose cheap fast foods, whereas Cluster 3 students may have benefited from more stable schedules and campus offerings, or simply the knowledge and motivation to seek healthier choices. Furthermore, gender differences in our results (albeit subtle) are supported by literature—female students in some studies report different coping strategies (like healthier food choices or seeking social support) compared to males (17, 18), which can influence both diet and academic resilience. Overall, our study’s profiles and the recent literature converge on a crucial point: multidimensional interventions are needed to address these intertwined issues.

From a public health and educational policy perspective, these findings carry several implications. First, identifying high-risk student clusters (like our Cluster 1) enables targeted intervention. Universities and health authorities should prioritize support for students who are socioeconomically disadvantaged and nutritionally at risk. This could include expanding campus food security programs (such as subsidized cafeterias or food pantries), providing meal plans or vouchers for low-income students, and ensuring that healthy, affordable food options are readily available on or near campus (2). Such measures directly address the economic barriers to good nutrition. At the same time, educational interventions can be deployed: nutrition literacy and cooking workshops could be offered to improve students’ ability to prepare low-cost healthy meals. Embedding nutrition education into university orientation or first-year programs may help instill better eating habits early on. Importantly, the success of these interventions may depend on pairing them with psychosocial support. High stress levels and mental health struggles were implicit factors for the low-performing clusters; therefore, universities should integrate mental health services and stress-management resources as part of student wellness programs. Initiatives like peer mentoring, academic counseling, and time-management training can alleviate the academic pressures that often drive poor eating and burnout (19–21). Indeed, recent recommendations emphasize a holistic approach—for example, one multination study concluded that campus strategies must be adapted to local context but generally should combine nutrition education, physical activity promotion, and psychosocial support to effectively improve diet quality and student health. Our results strongly support this comprehensive strategy. Students in Cluster 3 thrived likely because they had both the knowledge and the environment to maintain healthy lifestyles; replicating these conditions for other students is a key goal. Educational policy could also recognize nutrition as a component of academic success. Just as tutoring or financial aid are provided to boost academic outcomes, access to healthy food and lifestyle coaching could be viewed as investments in academic performance. This study adds to a growing body of evidence that academic institutions should treat student nutrition and wellbeing as integral to their academic mission (22, 23).

In interpreting our findings, it is important to acknowledge the study’s strengths and limitations. A major strength is the use of MCA and cluster analysis, which allowed us to uncover latent groupings and multidimensional relationships that conventional single-factor analyses might miss. By considering many categorical variables simultaneously, we captured the complex interplay of social, dietary, and academic factors in a data-driven way. Few prior studies have applied such techniques in this context, so our approach provides novel insights that complement existing regression-based evidence. The identification of clear clusters (e.g., a low-SES/low-performance cluster) provides a concrete basis for comparison with similar multivariate profiles reported in literature (such as latent class analyses of student health behaviors). However, our study is cross-sectional, which limits causal inference. We cannot conclusively say, for example, that poor diet causes poor grades or vice versa—likely the relationship is bidirectional and influenced by third factors like stress. Longitudinal research would be valuable to track how changes in social or nutritional status translate into academic trajectories. Another limitation is that our sample is regionally specific (university students in Ecuador), and while it shares many characteristics with student populations elsewhere in Latin America, caution is needed in generalizing globally. Cultural factors (dietary customs, education systems) can modulate these relationships. Nonetheless, the consistency of our findings with international studies (from North America, Europe, etc.) suggests that many observed patterns are broadly applicable (24, 25).

We also relied on self-reported data for measures like dietary habits and academic satisfaction, which can introduce response bias. Participants may have under- or over-reported certain behaviors. To mitigate this, we used validated survey instruments, but some error is inevitable. Future studies might incorporate objective measures (e.g., GPA records, nutritional biomarkers) for more precision. Despite these caveats, our results are robust in highlighting key clusters of determinants that matter for student health and achievement.

This discussion illustrates that university students’ nutritional behaviors and academic performance are deeply interconnected through a web of social determinants. Family environment, economic resources, and psychosocial wellbeing collectively shape whether a student falls into a high-risk or resilient profile. Our use of MCA and clustering has provided a nuanced mapping of these profiles, reinforcing findings from recent literature that call for integrated solutions. Public health and educational stakeholders should view student nutrition not in isolation, but as a crucial piece of the academic success puzzle. By addressing social inequities (like food insecurity and financial stress) and promoting healthy lifestyles on campus, institutions can improve not only students’ health outcomes but also their educational attainment.

This aligns with emerging multivariate studies and the broader social determinants framework: healthier, well-supported students are more likely to become high-performing, satisfied graduates. Finally, further research—using approaches such as latent class analysis or longitudinal clustering—should extend these insights, exploring how changes in social or nutritional factors over time impact academic trajectories. Such work will continue to inform interventions that ensure all students, regardless of their background, have the opportunity to thrive both in their diet and their education.

Beyond the institutional context, the findings of this study reflect a broader reality observed in many universities across developing countries, including Mexico and other Latin American settings, where social inequalities, food environments, and academic demands interact to shape students’ nutritional behaviors. This convergence highlights the relevance of generating coordinated public health and educational policies that transcend national boundaries and address nutrition as a structural determinant of academic performance (7, 26).

Additionally, the post-pandemic context has introduced persistent changes in dietary habits among university students. Disruptions caused by the COVID-19 pandemic—such as increased reliance on ultra-processed foods, irregular meal schedules, reduced physical activity, and heightened psychosocial stress—have continued to influence eating behaviors even after the return to in-person academic activities. These post-pandemic sequelae may exacerbate pre-existing social and nutritional inequalities, reinforcing the need to interpret current dietary patterns within a broader temporal and contextual framework (27, 28).

## 5 Limitations

This study has several limitations that should be considered when interpreting the findings. First, the use of self-reported instruments may introduce information bias, including recall bias and social desirability bias, particularly for variables related to dietary habits, living conditions, and perceived academic performance. Although validated questionnaires were used and data collection was conducted anonymously to reduce reporting bias, some degree of misclassification cannot be completely ruled out.

Second, the cross-sectional design precludes causal inference. The observed associations between social determinants, nutritional behaviors, and academic performance reflect concurrent patterns rather than directional or temporal relationships. Future longitudinal studies are needed to examine causal pathways and changes over time.

Third, the study was conducted in a single public university, which may limit the external validity and generalizability of the results to other academic institutions or student populations with different sociocultural, economic, or educational characteristics. Nevertheless, the institutional context provides valuable insights into the interactions between social determinants, nutrition, and academic performance in a Latin American university setting.

Despite these limitations, the study’s strengths include the use of advanced multivariate techniques, such as Multiple Correspondence Analysis and cluster analysis, which allowed the identification of latent patterns and heterogeneous student profiles that may not be captured through conventional analytical approaches.

## 6 Conclusion

This study provides empirical evidence that nutritional status and academic performance among Ecuadorian university students are not isolated outcomes but rather the product of interacting social determinants. By applying Multiple Correspondence Analysis (MCA) and cluster analysis to a multidimensional set of sociodemographic, nutritional, and academic variables, we identified distinct student profiles that reflect systematic inequalities in living conditions, food access, dietary behaviors, and perceived academic performance.

The results demonstrate that socioeconomic context and family background are central in shaping students’ nutritional practices, which in turn are associated with academic outcomes. Regular breakfast consumption, better food access, and more favorable socioeconomic conditions consistently clustered with higher academic satisfaction and performance. Conversely, students reporting financial constraints, food access difficulties, and less structured dietary habits tended to cluster into profiles characterized by lower academic satisfaction. These findings confirm that nutrition functions as a mediating pathway through which social determinants influence educational attainment.

Methodologically, the use of MCA and clustering proved effective in uncovering latent structures and heterogeneous profiles that traditional regression-based approaches may overlook. The identification of four interpretable clusters highlights the heterogeneity of student experiences and underscores the need for differentiated institutional strategies rather than uniform interventions.

From a public health and higher education perspective, the findings support the integration of nutrition and social support policies into academic success frameworks. Universities should prioritize targeted actions for students in socially and nutritionally vulnerable profiles, including food security programs, nutrition education, and psychosocial support services. Such multidimensional interventions have the potential not only to improve health outcomes but also to enhance academic engagement and performance.

Future research should adopt longitudinal designs to explore causal pathways and evaluate the effectiveness of policy interventions aimed at reducing nutritional and educational inequalities within university populations.

## Data Availability

The original contributions presented in the study are included in the article/supplementary material, further inquiries can be directed to the corresponding author.
